# Adolescents’ life between violence and discipline. Medical care in the “Jugendhäuser” juvenile detention centers in East Germany in the 1960s–1980s

**DOI:** 10.3389/fpubh.2024.1288025

**Published:** 2024-01-29

**Authors:** Oxana Kosenko, Florian Steger

**Affiliations:** Institute of the History, Philosophy and Ethics of Medicine, Ulm University, Ulm, Germany

**Keywords:** medical care, juvenile prisons, violence, suicide, self-harm, tattoos, East Germany

## Abstract

**Background:**

In 1952–1989, special juvenile detention centers, called Jugendhäuser, were established in the German Democratic Republic. There, juvenile delinquents had to not only complete their sentences, but they were also supposed to be re-educated into conscious socialist personalities through a system of collective education proposed by Soviet pedagogue Anton Makarenko. Among twelve Jugendhäuser in East Germany, the ones in Halle and Dessau were considered to have the most severe conditions due to the praxis of mental and physical violence. For the first time, based on the personal files of former juvenile prisoners and archival documents of medical services, we reconstruct a picture of the health status of prisoners and medical care in both these Jugendhäuser.

**Methods:**

We analyzed personal files of juvenile prisoners from the Archive of the Correctional Facility in Halle and unpublished documents from the Saxony-Anhalt State Archive, Magdeburg Department, the State Archive in Leipzig and the Stasi Records Archive in Halle. For the examination of these sources, we implemented the historical-critical method.

**Results:**

The Jugendhäuser had a system of outpatient and inpatient treatment. Although the medical services rated the level of health care as good at those detention centers, numerous complaints from juveniles, as well as cases of failure to provide assistance, indicated certain deficiencies. Cases of violence in juvenile prisons were common, especially in the Jugendhaus Halle. Brawls between inmates led to injuries and sometimes even to deaths. Fear of beatings resulted in desperate acts such as self-harm, suicide and escapes from prison.

**Conclusion:**

The health status of young prisoners in the Jugendhäuser in Halle and Dessau was negatively affected by violence and often by lack of medical care. The prevalence of violence can be attributed to challenges of the penal system as well as deficiencies of the medical services. Since repressive means were used to overcome the violence, such efforts were not successful. The medical services did not offer specialized care for juveniles with mental and learning disorders or those who required psychological or even psychotherapeutic support. Physical health issues were also often ignored due to the stigmatization of sick juveniles as malingerers.

## 1 Introduction

**Figure 1:**
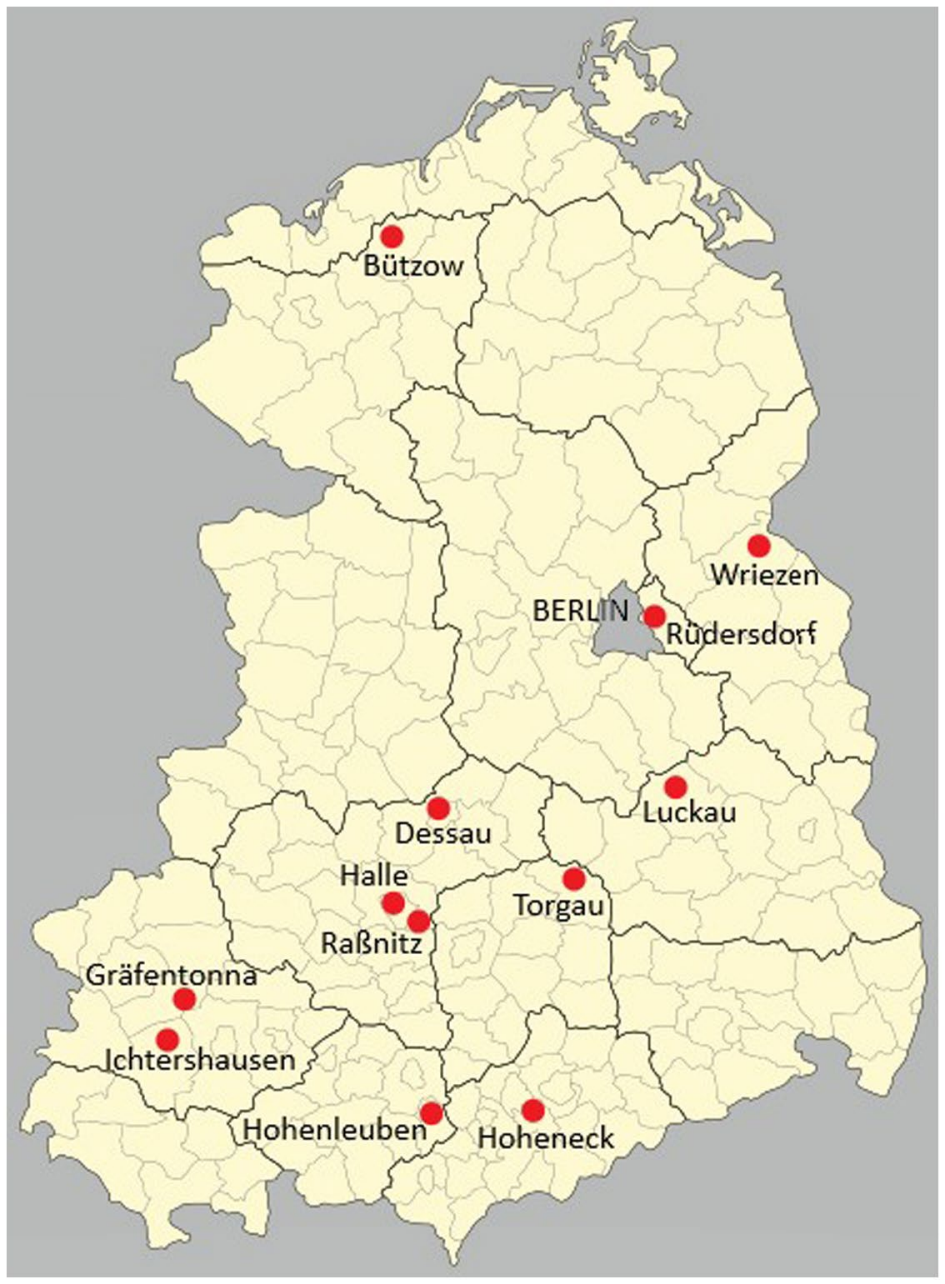
Jugendhäuser in East Germany.

“Jugendhaus” was the official name of a juvenile detention center or a youth prison in the German Democratic Republic (GDR). Jugendhäuser were established based on the Juvenile Courts Act of 1952. Their purpose was to ensure that adolescents between 14 and 18 years of age were held separately from adults. Initially, young prisoners were sentenced to variable prison terms of one to three years. They did not know how long their sentence would be, but they were obliged to serve at least a year in jail. This uncertainty was considered an effective method of forcing young people to behave properly. The 1977 penal reform abolished variable sentences (1). It is necessary to mention that the Jugendhäuser are often confused with the so-called “youth work yards” or “Jugendwerkhöfe.” A Jugendwerkhof was a special home for juveniles who were classified as difficult to educate. Although it had a repressive character, it was not considered a juvenile penal institution, but officially belonged to the youth welfare system (2). According to various sources, which we have summarized, there were about twelve Jugendhäuser during the entire existence of the GDR (Figure 1). In each of five GDR states there were from one to three Jugendhäuser (Figure 1). One Jugendhaus was established in Saxony, in Torgau. Mecklenburg had another one in Bützow. In Saxony-Anhalt, three Jugendhäuser were founded: in Dessau, Halle and in Raßnitz. The Jugendhäuser Luckau, Rüdersdorf and Wriezen were located in Brandenburg. The other ones in Thuringia were founded in Ichtershausen, Gräfentonna and Hohenleuben. The last one was intended for the detention of delinquent girls (3). From 1965, some female juveniles from the Jugendhaus Hohenleuben were transferred to Hoheneck Women’s Prison. This was the largest female prison in the GDR with a section for adolescents (4). Thus, the female juvenile prisoners were detained separately.

**Figure 2:**
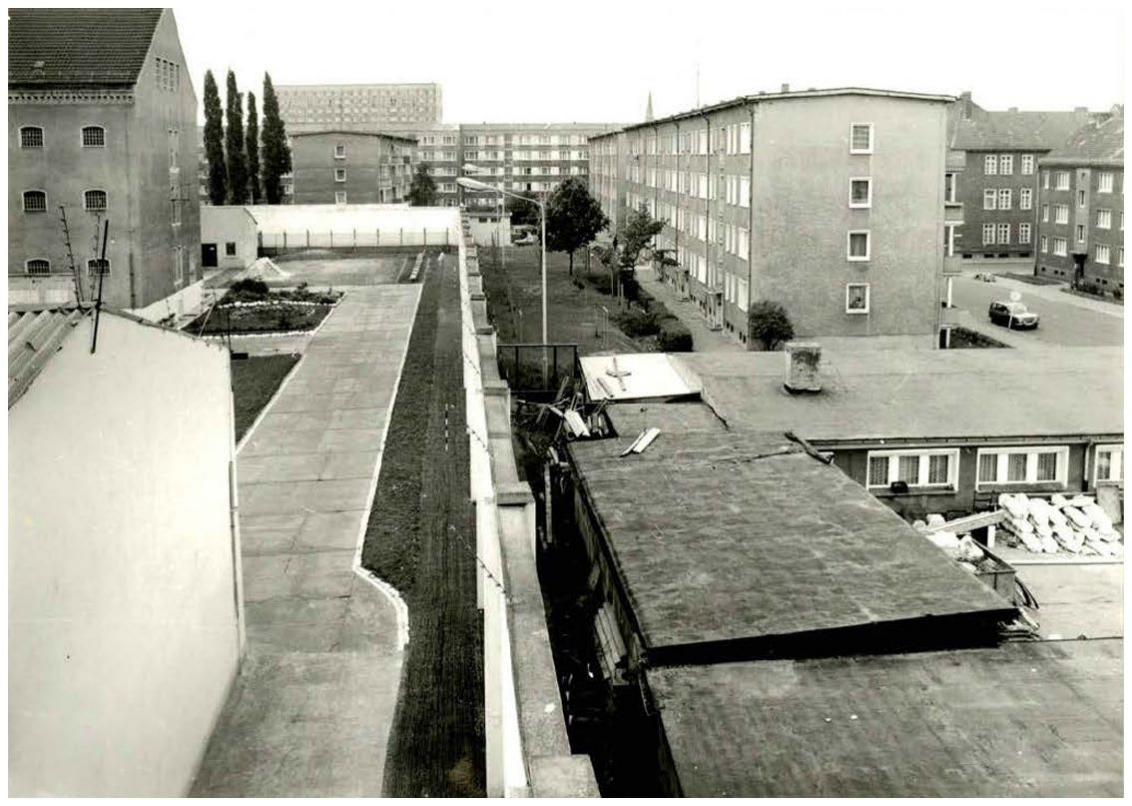
View from the second observation tower to the west side of the defense wall of the Jugendhaus Dessau. German Federal Archives, Stasi Records Archive in Halle. BArch, MfS, BV Halle, AG XXII, Nr. 269, BStU S. 15. Reprinted with permission from Bundesarchiv, © Bundesarchiv.

**Figure 3:**
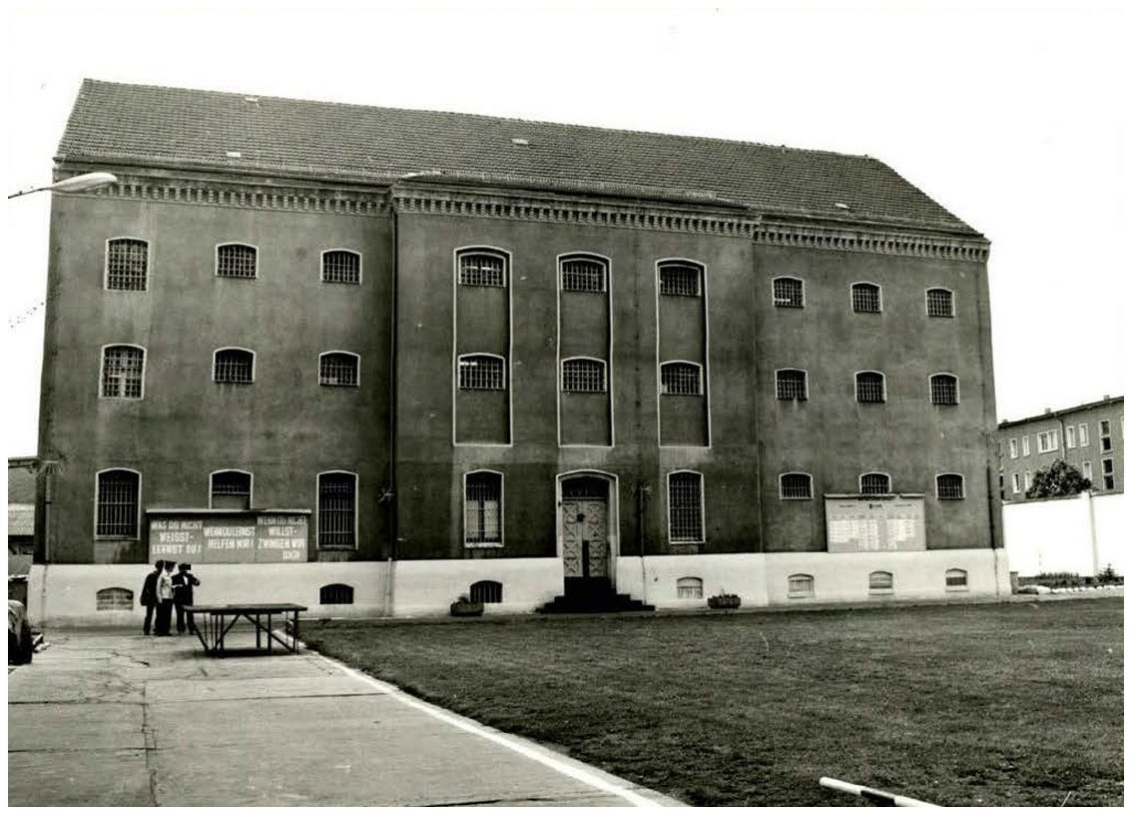
North side of the detention facility 1 of the Jugendhaus Dessau. German Federal Archives, Stasi Records Archive in Halle. BArch, MfS, BV Halle, AG XXII, Nr. 269, BStU S. 27. Reprinted with permission from Bundesarchiv, © Bundesarchiv.

**Figure 4:**
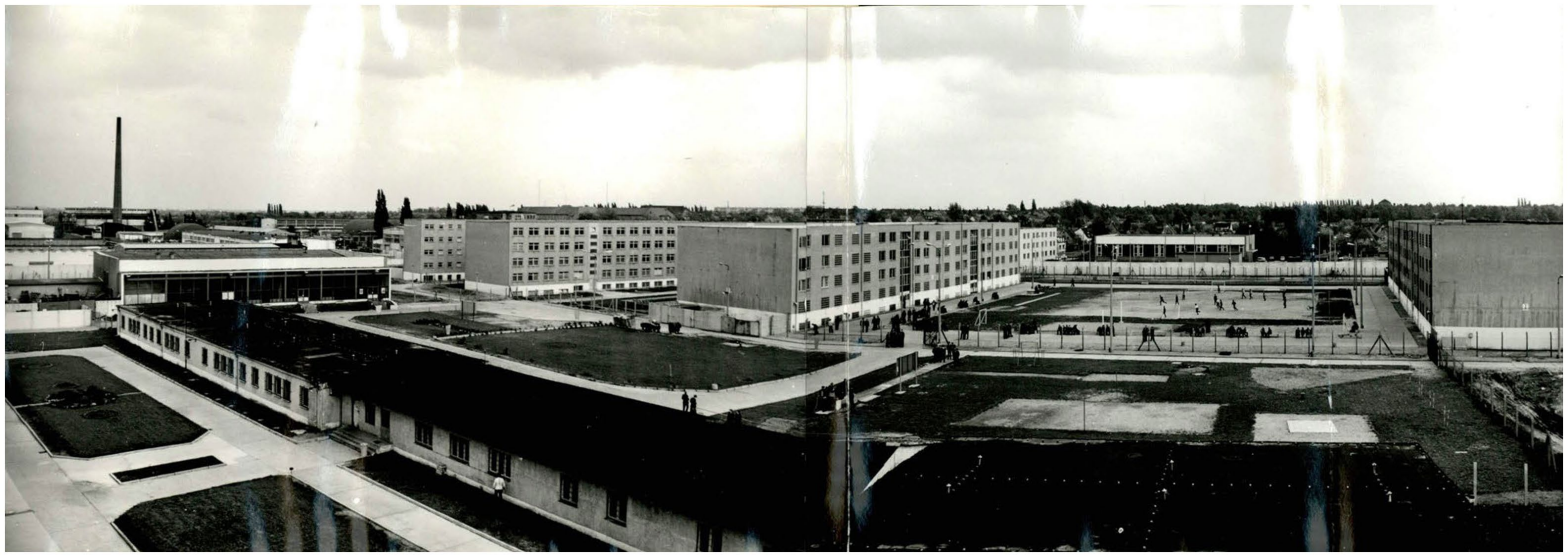
General view of the Jugendhaus Halle seen from north to south. German Federal Archives, Stasi Records archive in Halle. BArch, MfS, BV Halle, Abt. VII, Nr. 831, BStU S. 1. Reprinted with permission from Bundesarchiv, © Bundesarchiv.

The juvenile detention centers in Halle and Dessau were considered particularly notorious places due to the systematic use of psychological and mental violence. The Jugendhaus Dessau was founded in 1952 (Figures 2, 3) and the one in Halle (Figure 4) in 1971 (1). The holding capacity of the Jugendhaus Dessau was of 800 juveniles. Due to the amnesty of 1979, the number of juvenile prisoners was reduced, so the Dessau contractual companies could not be sufficiently supplied with workers. Therefore, adult prisoners were allocated from other prisons to the Jugendhaus Dessau. In 1981, there were 258 juveniles of the age between 14 and 18 years and 126 adults (5). The Jugendhaus Halle was one of the most modern penal facilities of its time in the GDR. Initially, it was built with a holding capacity of about 1,200 adolescents. As a part of the centralization of juvenile prisoners, the construction of two more buildings took place in the Jugendhaus Halle, increasing its capacity to 1,800 juveniles in 1981. The majority of the juveniles in the Jugendhaus Halle were political prisoners sentenced under paragraphs 213 (unlawful border crossing) and 215 (hooliganism) of the Penal Code of the GDR (6).

According to the Juvenile Courts Act of 1952, the goal of the Jugendhäuser was to re-educate adolescents who were still considered capable of self-improvement. However, this mission statement was largely disregarded by the authorities of the Jugendhäuser, who believed that educational measures were to be carried out in Jugendwerkhöfe, while punishment was to happen in the Jugendhäuser (2). However, according to the regulations of the GDR penal system, educational success was to be achieved through socially useful work, vocational qualification measures, civic and general education, cultural and sports activities, enforcement of order and discipline, and a system of self-education. Following the model of collective education of Soviet pedagogue Anton Makarenko (1888–1939) (7, 8), the prisoners were divided into groups, for each of which a so-called “educator” was responsible. There was a system of competition between groups for the best discipline, order and work achievements. The best collectives were rewarded, which encouraged the juveniles to ensure that everyone complied with the fulfilment of the norms. However, an educator delegated some of his authority to selected juveniles, but these often overstepped their authority and used violence against other members of the group. The prisoners at the bottom of the hierarchy were subjected to harassment, psychological or physical torture and sexual assault. The educators often tolerated those violent excesses. The penal system did not provide for a separation of political and criminal prisoners. Juveniles with mental disorders and diseases were also detained together with other young delinquents (1, 2).

The history of Jugendhäuser has not been sufficiently researched. In comparison to it, a more detailed analysis has been written about Jugendwerkhöfe (2, 9). The research on the Jugendhäuser is limited to Halle and Dessau, in which the main topic is political persecution (1, 3, 10). The researchers mostly only briefly touch on the aspect of medical care for adolescents, with their main sources of information being statements of former juvenile prisoners. In general, it should be noted that in research works on correctional facilities, the medical aspect of detention is rarely the subject of special investigation. First research attempts in this direction were made in the case of the Hohenschönhausen prison, the main political prison of the former East German Communist Ministry of State Security (11–13). This prison was notoriously famous for detaining almost all known GDR oppositionists, who were physically and psychologically tortured there. Worse still, the doctors of the prison hospital worked not for the benefit of patients, but on behalf of the Ministry of State Security. The therapy goal was not to restore the prisoners’ health, but to create the conditions for them to give the necessary testimony. Therefore, a research should be undertaken in this direction in order to provide a picture of the detention and medical service in prisons of the GDR.

Thus, the reconstruction of the most objective picture on the conditions of medical detention in prisons is only possible with the involvement of archival documents of penitentiary facilities, their medical services, and, most importantly, by consulting the personal files of prisoners themselves. The latter present the greatest challenge in relation to the protection of the personal data of former prisoners. Moreover, in our case, the personal files of former juvenile prisoners are stored in the ongoing archive of the Correctional Facility in Halle. It is usually not easy to gain access to the materials held in archives kept by an administrative authority, especially if the information is sensitive. It has therefore been a long and laborious process to obtain this information. The State Commissioner for the Study of the Communist Dictatorship in Eastern Germany for the state Saxony-Anhalt, Birgit Neumann-Becker, and the Ministry of Justice of Saxony-Anhalt are to be thanked for their support in making these files accessible for our research for the first time. Besides the evaluation of the personal files of the former juvenile prisoners, we also examined the records of the medical services of both Jugendhäuser. These sources have enabled us to reconstruct a picture of the health status of prisoners and of their medical care.

We structured our paper as follows: firstly, we focus on the organization and work of the medical services in the Jugendhäuser Dessau and Halle. Next, we provide information on the abuse of prisoners and examine cases of self-harm, suicides, escapes and refusal to eat which were a form of protest against violence. In the discussion, we compare these results with the experiences provided by the juvenile prisoners themselves and evaluate the organization of medical services as well as the health state of juveniles in both these Jugendhäuser.

## 2 Materials and methods

We analyzed personal files of the former juvenile prisoners from the Jugendhäuser Dessau and Halle stored at the Archive of the Correctional Facility in Halle and unpublished documents from the Saxony-Anhalt State Archive, Magdeburg Department (Landeshauptarchiv Sachsen-Anhalt, Abteilung Magdeburg), the State Archive in Leipzig (Staatsarchiv Leipzig) and the Stasi Records Archive in Halle (Stasi-Unterlagen-Archiv Halle), which is part of the German Federal Archives (Bundesarchiv). The Archive of the Correctional Facility in Halle holds a total of about 30,000 personal files of juvenile prisoners, of which about 15,000 files of the Jugendhaus Halle from 1972–1990 and around 15,000 files of the Jugendhaus Dessau from 1961–1962 and 1971–1990. Of those 30,000 files, we evaluated about 1,200 files from different years. Since we did a qualitative and not quantitative research, our size sample depended on saturation or “informational power” (14, 15) which we reached by 1,200 files. This means that we identified those personal files that contained the most complete information about the health condition of adolescents. Since most of the personal files contain only a standard medical examination form, which includes anamnestic, social and diagnostic data, our searches were focused on more detailed information about adolescents’ diseases and their health condition, such as: medical histories, hospital referrals and discharges, medical reports, psychiatric reports on juveniles with violent or suicidal tendencies, information about tattoos, chest radiographs and “weight record cards,” in which the weight of the adolescents was to be registered each month. Hospital referrals were particularly informative, because they indicated the patient’s condition upon admission and often revealed the cause of a disease or injury. Hospital discharge summaries and medical reports often contained not only the information about the patients’ condition, but also their behavior during their stay in hospital, such as suicide attempts or self-injury.

The Saxony-Anhalt State Archive contains important records of the medical services of both Jugendhäuser, such as minutes of consultations, work plans, monthly and quarterly reports on the state of health of juveniles. The records of the medical services in the Jugendhäuser in Halle and Dessau have not been preserved intact. However, the records of the Jugendhaus Dessau for the years 1962–1983 provide a sufficient insight into the work of the medical service. The archive also holds records of the District Authority of the German People’s Police of Halle, which contain correspondence about the work of the prison health services. This information is especially valuable because it reveals the actual situation in the Jugendhäuser. Specifically, these documents include complaints from adolescents about medical care or violence by cellmates or prison staff, as well as information about emergency incidents such as murders of inmates or their escapes. We also analyzed documents of the prison hospital in Leipzig, stored at the State Archive in Leipzig, as juveniles were often taken to that hospital for inpatient treatment. The hospital documents primarily reveal hygiene violations and lack of medical staff. The Stasi Records Archive in Halle, which is part of the German Federal Archives (Bundesarchiv), stores documents of the Halle district administration of the Ministry for State Security. The Stasi main department VII was responsible for counterintelligence in the Ministry of the Interior and the German People’s Police. Therefore, we could find some information about the situation and about some incidents in the Jugendhaus Halle in the department’s documents.

In our paper, we did not include interviews with former juvenile prisoners. The available research on the topic already includes published interviews (1, 3, 10). These were largely the main information sources on the detention of adolescents in Jugendhäuser for researchers who were unable to access medical records of the juveniles. Moreover, since eyewitness recollections, especially about their traumatic experiences, belong to the category of subjective sources, in our paper we strived to verify them using archival sources.

To examine these sources, we implemented the historical-critical method, which includes the stages of acquisition of primary sources and research works, critical evaluation of the information contained in the primary sources, and presentation of historical data in the historical context in terms of objectivity and significance (16).

## 3 Results

### 3.1 Medical service and treatment of diseases

**Figure 5:**
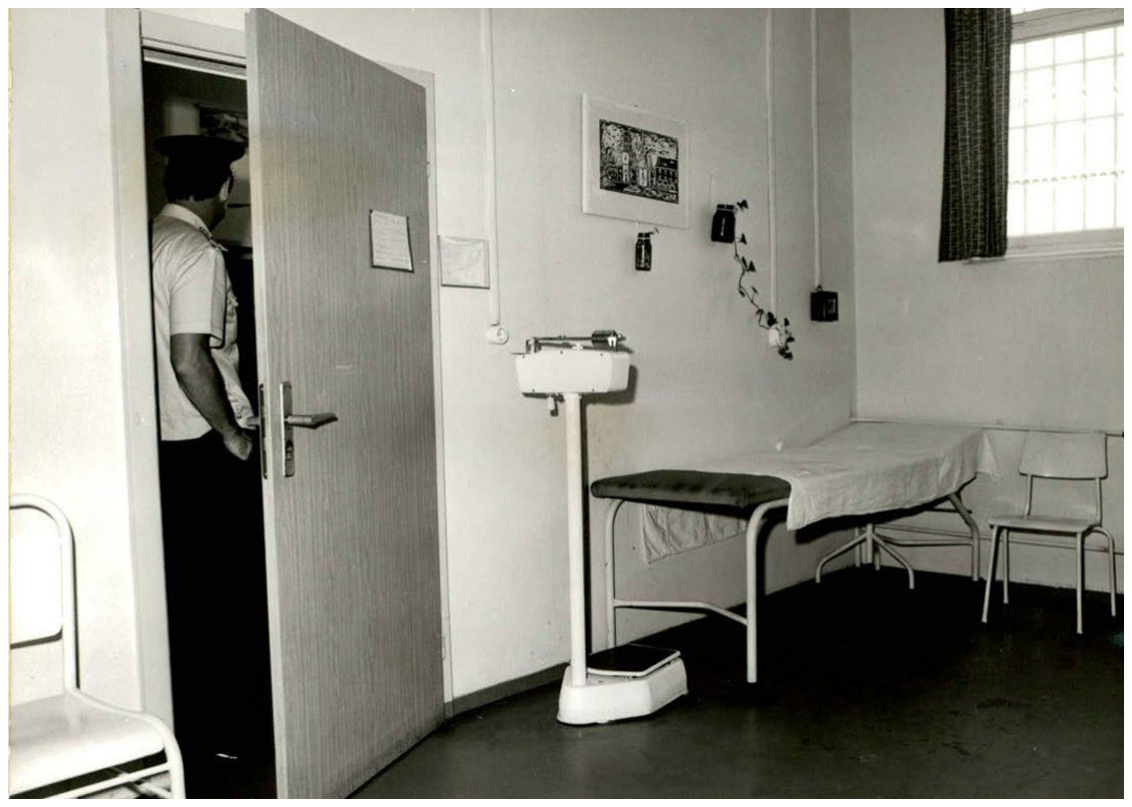
Anteroom to the treatment room of the medical service of the Jugendhaus Halle. German Federal Archives, Stasi Records Archive in Halle. BArch, MfS, BV Halle, Abt. VII, Nr. 831, BStU S. 89. Reprinted with permission from Bundesarchiv, © Bundesarchiv.

The Jugendhäuser had a system of outpatient and inpatient treatment. The outpatient treatment was carried out either in the first-aid rooms (“Sanitätsstuben”), used for the examination and therapy of slightly ill juveniles who were fit for work, or in the outpatient departments (“Ambulanzen”) for preventive prophylaxis, diagnostics, therapy and aftercare (Figure 5). Inpatient care was also provided directly at the Jugendhaus. The infirmaries (“Krankenreviere”) were used for the accommodation of slightly ill or injured juveniles who were temporarily incapacitated and did not require permanent medical care or treatment. At the beginning of the 1960s, an infirmary in the Jugendhaus Dessau had a capacity of approximately 120 patients (17). If juveniles needed specialized inpatient medical therapy and permanent medical care, they were sent to a prison hospital. That was necessary, for example, in case of surgical operations such as appendix or tumor removals. Adolescents were often sent to the hospital for emergency treatment of injuries, like fractures or head injuries resulting from fights between inmates.

To improve medical care of juvenile prisoners, agreement contracts were signed with the Dessau District Hospital and policlinics. Due to these contracts, doctors from the hospital and policlinics had visiting hours in the Jugendhaus. By providing many medical examinations within the prison itself, this saved the cost of transporting sick prisoners and avoided the need to have them escorted by operational forces (18). Juvenile detention centers also included a dental station. From 1977, the medical service had an X-ray machine at its disposal and took thorax X-rays of juveniles. The evaluation of X-ray images was carried out by a tuberculosis district doctor (19). The staff of the medical services was not only responsible for consultations and treatment of patients, it was also in charge of security and order in the infirmaries, conducted weight and hygiene checks on all juveniles, inspected the hygienic condition of the rooms, kitchen and sanitary facilities. In the 1960s, there was a contracted doctor who offered consultations four times a week, and in case of sudden illnesses he was also available outside of fixed consultation hours. The dentist held consultations twice a week and was also available outside scheduled hours (20). The medical service in the late 1970s consisted of 8–9 members of staff (21).

The most common diseases of juveniles reported by the medical services were influenza, tonsillitis, skin diseases and upper respiratory tract infections. The juveniles also received medical care after accidents, brawls with fellow prisoners, and after a stay in solitary confinement (21). Among the diseases that were treated in a prison hospital were appendicitis, pneumonia, fractures. If doctors could not diagnose a juvenile or did not have the technical capacity for that, he was referred to a specialist, often to the University Clinic of Halle. Such cases occurred, for example, for skin diseases (eczema or psoriasis) or gastrointestinal diseases (stomach ulcer).

Reports from the health services either do not mention the state of health care or rate it as good (22). However, if we look at the reports of the head of the Jugendhaus Halle, we constantly find complaints of juveniles about poor medical care or refusal by the doctors to refer to a specialist. It is often mentioned that parents of juveniles expressed their concerns about their children’s health (23). One visually impaired juvenile who was dissatisfied with medical care even wrote a complaint to the Ministry of Health of the GDR. However, his letter was intercepted (24). There also was a case when a juvenile required immediate medical help, which was not provided promptly. In 1974, in the Jugendhaus Halle, a prisoner was beaten to death by another inmate. As the autopsy on the adolescent’s body revealed, if medical assistance had been provided by the medical service within the first three hours, the juvenile would have survived. The prison authorities stated that medical services declined to provide many treatments because they often categorized the juveniles as malingerers (25).

When it came to juveniles with mental or developmental disorders, they did not receive any special treatment. For example, in the case of a 16-year-old prisoner from the Jugendhaus Halle, who was diagnosed with an intellectual disorder as well as hypersexuality, sedatives in the form of phenobarbital were administered (26).

### 3.2 Violence and its consequences

As we can see from the archival documents, fights between fellow inmates were a constant problem in the juvenile detention centers. However, this problem drew the attention of prison authorities only when particularly severe cases occurred. For example, in the Jugendhaus Halle, 17 cases of brawls were reported in the first quarter of 1962 alone (27). Brawls were often so violent that prisoners needed about a week to recover (28) or had to be sent to a hospital due to the severity of injuries. Some juveniles were referred to the hospital for examination multiple times as a result of repeated beatings. Most often, they were hit in the face or slammed against a wall with the back of their heads, which caused cerebral contusion (29).

The brawls were sometimes even lethal. One such aforementioned case took place in the Jugendhaus Halle in 1974, when one juvenile beat another inmate to death. The autopsy findings showed that the juvenile died due to cerebral hemorrhage caused by multiple blows to the face. The main concern of the authorities of the juvenile prison in this regard was that the information about this case might fall into the hands of Western agents. The prison authorities were afraid that should this occur, they would not be able to deny that the socialist penal system was different from the capitalist one, although they believed that re-education of GDR citizens who had committed crimes was based on completely different principles. The prison authorities wondered why a murderer had been appointed a group leader, even though he was already known as a bully and a boxer. They recorded the case into his personal file. This would have led to an additional sentence and to the exclusion of the possibility of early release. However, nothing was done about the educator who appointed the murderer as a group leader and probably did nothing to prevent his violence against other inmates. According to the logic of the prison authorities, it was not a question of finding out who did something wrong, because the causes would have been found exclusively at the management level (30). Therefore, there were no sanctions against the educator, but his supervisor and department head was removed from his position. In order to prevent brawls, the following measures were introduced: tightening of the prison regime, approval of a team leader from the head enforcement service, equipping operational forces with police batons, and full occupation of the juveniles’ non-working time (31). However, these measures did not help. A month later, the authorities of the Jugendhaus Halle had to state that the brawls just did not stop (30). The reports of the head of the Jugendhaus Halle also mentioned cases of assaults committed by prison staff against juveniles (32) and unacceptable disciplinary methods used by the educators, such as “Duck-Walk” and “Wheelbarrow” exercises (33). Fear of being beaten even led some juveniles to desperate solutions: escape, self-harm or suicide.

#### 3.2.1 Escapes

A few escapes happened in 1972 at the Jugendhaus Halle. In one case, a 19-year-old prisoner was appointed leader for a group that carried out shaft work at a school in Halle. He was chosen because he showed good order and cleanliness, as well as cooperation in group lessons. His task was to assign 13 juvenile prisoners in his group according to the work to be done. During the allocation of tasks, a conflict arose between the team leader and other inmates who threatened to beat him up later in the detention house. He was so certain that he would be caught and beaten that he found no better solution to his problem than to escape from detention (34). In another case, a juvenile prisoner was constantly beaten and escaped as he could no longer withstand the abuse from his inmates. The reason for the bullying of the 16-year-old boy was that he was a “bed-wetter.” Apparently, the prisoner had some developmental problems as it was mentioned that he was socially inhibited and his mental development was below the level appropriate for his age (35). Both escapees were caught and brought back to the Jugendhaus Halle.

#### 3.2.2 Self-harm and suicides

If we open any of the medical examination forms in the personal files, we notice some questions that were to be answered at the discharge examination of an adolescent. For example, did the prisoner suffer accidents or illnesses during the execution of the sentence that limited his capacity? Did the prisoner attempt suicide or self-harm or swallow foreign objects? The very posing of these questions indicates that such cases were common.

As we can see from the documents, under such strict conditions of control and discipline, prisoners had limited opportunities to commit suicide. Thus, the most common method of suicide was by swallowing foreign objects that prisoners would steal from the kitchen or from a workshop, such as tablespoons or screws. The following cases illustrate the desperate situation of juveniles. One juvenile who was often beaten and referred to the hospital in Leipzig at least two times, drunk some soldering fluid. However, the intoxication was successfully treated (35). Another young prisoner from the Jugendhaus Dessau attempted suicide several times in 1971. Initially, he swallowed a few screws. Luckily, they were excreted naturally. One month later, he had an accident at work and at the same time he had appendicitis, which was successfully operated. One more month later, he swallowed several foreign objects made of glass and metal (S-shaped bent 2-mm-thick wire, broken spoon handles, nails). He was immediately admitted to the Leipzig Prison Hospital for inpatient treatment. At first, he refused examination several times. Later he removed tattoos on his left hand with a piece of glass. The large bleeding wound was iodized and bound. Some glass fragments, probably from a drinking glass swallowed by the juvenile, exited his body naturally. A few days later, he ingested a large quantity of medication that he had allegedly stolen from the pharmacy. He was found lying in his bed in a comatose state. A gastric lavage was undertaken immediately. After a month of treatment in the prison hospital, he recovered and had to go back to detention. Shortly before the end of his hospital stay, he attempted suicide again by slitting his wrists. After his recovery one week later, he was transferred back to the Jugendhaus Halle (36).

In another even more insightful case in 1961 an inmate committed suicide, and in his farewell letter to his parents he clearly formulated the reason for his death: “It is absolutely senseless, under these circumstances, to keep on swelling for another 17 months, only to be released completely exhausted in health and nerves and unfit for any appealing work.” The 17-year-old strangled himself with a tablecloth. He had already spent a year in Dessau but realized that he would not be able to stand it any longer (37).

Refusal to eat as a form of protest and self-harm was also practiced in the Jugendhaus Halle. For example, this was the case in 1989 with some juvenile prisoners who were sentenced under § 213 (unlawful border crossing) of the Penal Code. Their refusal to eat and to work had two main reasons: their belief that they were unjustly imprisoned, as well as the beatings by prison staff. The juveniles also wrote letters to the General Secretary of the Socialist Unity Party of Germany, the Attorney General and the Supreme Court of the GDR requesting to review their cases (38). Unlike this incident of collective protest, there were also individual acts. One juvenile political prisoner managed to spread his negative attitude towards the policy of the ruling party and the government within his group. For this reason, he was temporarily segregated from his inmates, whereby his refusal to eat came into play as a demonstrative action (39).

### 3.3 Tattoos

**Figure 6:**
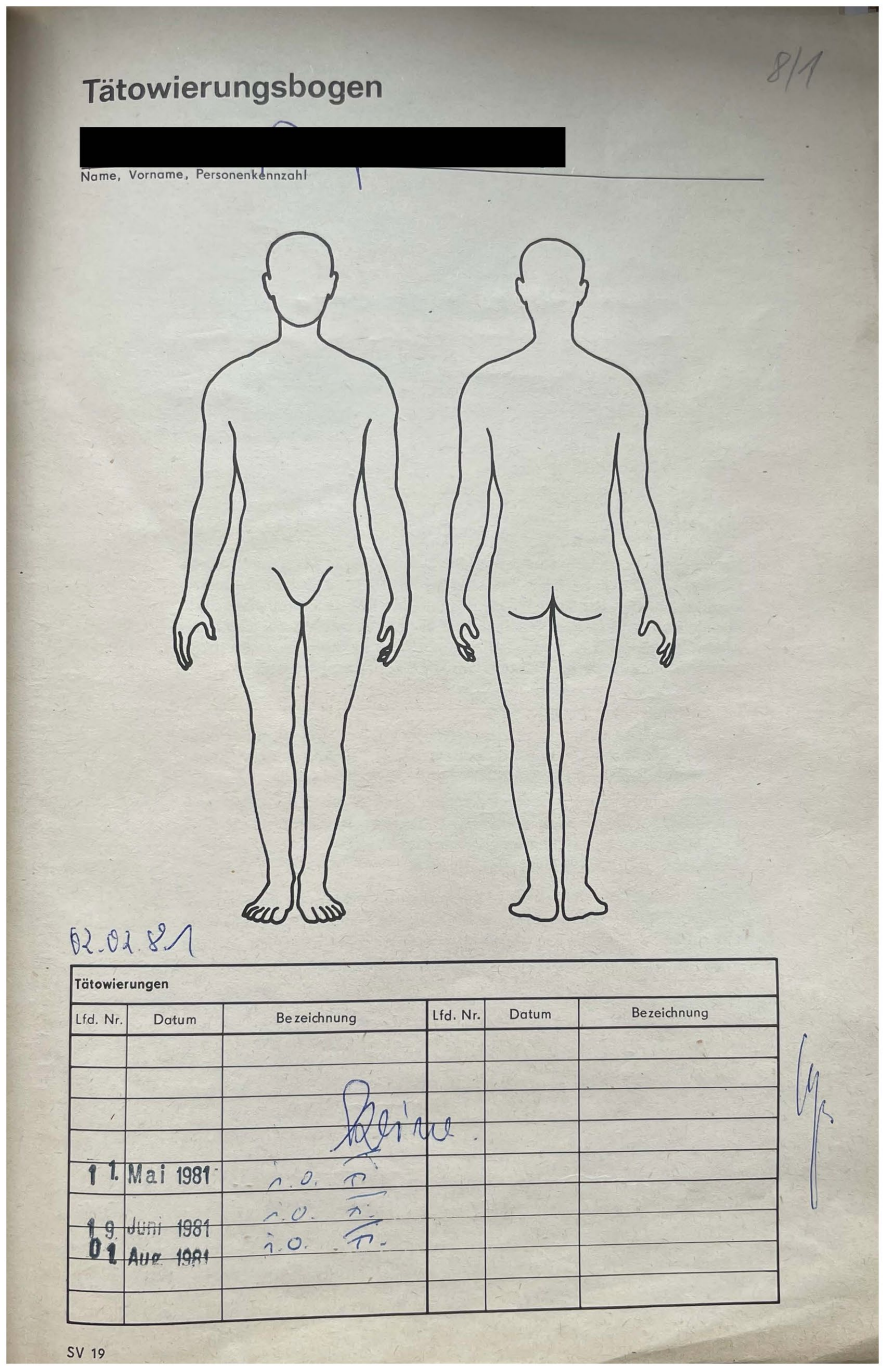
Tattoo form, 1981. Jugendhaus Halle. Archive of the Correctional Facility in Halle. Archive of the Correctional Facility in Halle. Reprinted with permission from Zentrale Auskunftsstelle des Justizvollzuges des Landes Sachsen-Anhalt bei der Justizvollzugsanstalt Halle, © Ministerium der Justiz des Landes Sachsen-Anhalt.

The control of tattoos was the responsibility of the doctors. Tattoos were checked regularly to see if new ones had been added (Figure 6). At the beginning of the 1970s, a tattoo questionnaire was added in personal files, and from the end of the 1970s an instruction on tattoos also appeared frequently. In parts of the text of this instruction, the ban on tattoos was justified exclusively on the grounds of the risk of developing skin cancer. For example, the instruction from 1977 and 1978 signed by a juvenile prisoner in the Jugendhaus Halle was formulated as follows: “According to specialists in dermatology (skin diseases), in evaluation of relevant cases, skin cancer can generally occur due to tattooing. To prevent such serious damage to health, it is therefore prohibited in the penal and pre-trial detention facilities to tattoo oneself or others or to get tattooed. Violators will be subject to disciplinary action” (40). As the text indicates, disciplinary measures should have followed in case of tattooing. However, the text of the instruction was soon supplemented in such a way that tattooing was not only subject to disciplinary, but also to criminal sanctions: namely, in cases when tattoos could “disparage the state order or state organs, institutions or social organizations or their actions” or “interfere with state activity or disregard the law in a way that endangers public order or have a military or fascist character” (41). In case of inmates found with such tattoos, which were made in the penal institution and were not removed or changed voluntarily, the application for a suspended sentence was generally rejected. In one instruction on tattoos from 1970 at the Jugendhaus Dessau it was stated that if the tattoos were fascist, military or state defamatory in character, they were to be surgically removed (42). It seems that in the late 1970s the prisoner’s consent was required to remove a tattoo. If he refused, it led to a penalty.

For example, in one case from 1979 tattoos with the inscriptions “Free – U.S.” and “lied, betrayed, raised to hate” with a swastika were to be removed from a prisoner’s body. However, after providing information about the possible consequences after the removal of the tattoos, the doctor could not convince the prisoner to remove them (43).

Nevertheless, some juveniles had tattoos done despite the risks and warnings. As some statistical reports show, tattooing was widespread in the early 1960s despite all prohibitions. According to a report from the Jugendhaus Dessau, 10 cases of tattooing were registered in the first quarter of 1962 (44). Individual cases also occurred in later years. For example, a young man from the Jugendhaus Dessau was tattooed with a tiger’s head on his right upper arm by a fellow inmate. For that, he received 3 days of solitary confinement. In his statement, he explained that he was using a refill of a ballpoint pen with a wire. He explained his decision as follows: “Actually I do not know why I got a tattoo, more out of boredom and silliness. Although I liked the picture too” (45).

## 4 Discussion

### 4.1 Challenges of the detention

As we can see from the results presented above, both the medical care and detention of juveniles faced a number of structural issues. The authorities of juvenile prisons relied on the Soviet experience of collective education for young people. However, Makarenko’s system, which encountered quite a number of positive assessments in various different countries, was implemented not quite in the way the founder of the pedagogical system himself had seen it. The educator of juveniles had to have a great moral responsibility in re-educating young people. High demands were made on his personal qualities: he must have great commitment, strong principles, balance, prudence, positive attitude to life and a sense of community (2). He also had to provide care and understand special needs of the individuals in his group. However, the educators seldom met these requirements, but they rather handled their duties formally. Not only did some of them fail to prevent the development of violence between inmates, they also abandoned their mentees to the mercy of fate or abused them themselves. In such a situation, it is impossible to speak about the educational function of the Jugendhäuser, which confirms the already mentioned opinion about juvenile prisons that their purpose was to punish adolescents, and not to re-educate them (2). The statements of former juvenile prisoners and members of the penal system about the violence prevailing in juvenile facilities found confirmation in the documents that we evaluated. However, incidents of violence were recorded in juvenile personal files only in cases of serious injuries or self-injuries, when juveniles needed to be sent to a prison hospital, or in cases of suicides. Former employees of the Jugendhaus Halle admitted that if the information about violence did not leak out, nothing was done to combat or prevent it. Only when violence led to serious consequences that could not be restrained, the abusers were punished (10). However, the prison staff did not want to destroy the hierarchy in the groups of juveniles, because it made their work easier (10). The documents that we evaluated did not mention sexual violence, but it was widespread in juvenile detention centers, as juveniles and prison members witnessed (1, 10). There is also some indication of sexual abuse of juveniles by educators. Juveniles with mental and learning disorders were especially bullied (10).

The authorities of the Jugendhäuser, as the experience of the Jugendhaus Halle shows, were unable to effectively eliminate the violence between inmates. After the aforementioned incident of the murder of a juvenile by his inmate in 1974, they resorted to repressive measures, such as tightening prison rules, equipping operational forces with police batons, and taking away the free time of juveniles. In the end, the juveniles not only did not get any benefit from these measures, but actually suffered even more. As a former employee of the Jugendhaus Halle confirms, until around 1977 the prison staff committed assaults on juveniles, often using police batons (10). The same can be linked to escapes from prison. Realizing that no one would help them, young people decided to take a desperate step, but upon being caught, they were to receive an additional sentence for violating the rules of the prison regime. We can also see that propaganda was of great importance in the ideological struggle with the West. The authorities of the Jugendhäuser needed to show that the delinquent youth were not just under control, but were being successfully re-educated into new socialist personalities. In reality, young people were left to themselves and they tortured each other. We assume that the situation in other juvenile detention centers was similar, but for confirming this assumption we have only fragmentary recollections of former adolescent inmates published on private websites. Obviously, further historical research is needed to create an overall picture of the detention of adolescents in the Jugendhäuser.

### 4.2 Health care issues and medical ethics

According to the former juvenile prisoners, access to medical services was often dependent on the goodwill of an educator. If the symptoms were not very noticeable or severe, juveniles did not always receive immediate medical examination (1). As we have seen in the 1974 case of the murder of a juvenile by his inmate, first aid was not always provided in emergency situations either. Moreover, medical services treated juveniles as malingerers who did not want to work.

The penitentiary laws of 1968 and 1977 of the GDR did not provide for social therapeutic support and individual treatment measures (46). Thus, specialized care for juveniles with mental or developmental disorders was completely lacking. Only in the 1980s a gradual understanding arose within the juvenile correctional system that such juveniles must receive special treatment (2). However, the Jugendhaus Halle, for example, also employed psychologists who were obviously not part of the medical service. According to the recollections of the psychologist R., who joined the Jugendhaus Halle in 1974, he worked in the intake department, where an educational program was developed for each juvenile. He noted that although the psychologists did not offer counseling to juvenile prisoners, as persons of trust at least they helped juveniles who were sexually abused. The victims were removed from the groups and assigned to new ones (10).

Speaking of suicides, it is to be noted that the suicide rate in the GDR prisons was lower than in West Germany. It can be explained by the fact that suicides of prisoners were prevented by constant supervision, arrest, or the administration of medication. If suicidal acts occurred, the security guards were threatened with disciplinary sanctions. In the Leipzig Prison Hospital, even lessons on suicide prevention were given to prison guards and officers (47). Indeed, as we have seen from the cases of juveniles who repeatedly attempted suicide in the Leipzig Prison Hospital and in the Jugendhäuser, their staff was able to prevent them timely. However, the problem was dealt with by repressive methods. As mentioned above, the juveniles did not receive any psychotherapeutic support either.

There were also a number of violations in the matter of medical ethics. As we could see from the presented cases of failure to provide medical care, especially in life and death situations, the doctors of the medical services violated the ethical principle of beneficence. Striking examples of this are also the suicide attempts, especially the repeated ones, which should have alerted physicians to the causes of suicidal behavior.

Finally, it should be noted that the prohibition of tattoos was not only enforced on the grounds of health risks and violation of the rules of the Jugendhäuser, but also for ideological reasons. If the image of a tattoo could insult the state authorities, it had to be removed from an adolescent’s body. Although the removal of a prohibited tattoo required the informed consent of the juvenile, in case he refused, he faced disciplinary actions. In modern Germany, tattooing is still forbidden in correctional facilities, which can be explained by health risks (infectious diseases, HIV) and disruptions in prisons. In must be added that tattoos are still an identification mark of prisoners and they are often forcibly inked by inmates. The ban therefore has its reasons (48). However, prisoners are not forced to remove tattoos anymore, especially not for ideological reasons.

## 5 Conclusion

The accounts of the former inmates and staff of the Jugendhäuser in Halle and Dessau of violence and lack of medical care find confirmation in the medical records of the prison authorities. The prevalence of violence in the Jugendhäuser in Halle and Dessau can be attributed to the challenges of the penal system itself and to the deficiencies of the medical services. The implementation of the educational system in practice resulted in cases of violence committed either by prison staff or by juveniles towards each other. The creation of hierarchy in groups of juveniles according to the system of collective education, as well as the lack of care from educators towards the oppressed, all contributed to the flourishing of violence. Attempts to combat it were not particularly successful because the prison authorities tried to solve the problem with repressive methods. While the prison medical services and hospitals were generally able to prevent suicides, they failed to address the problem *per se*. They did not offer any specialized care for juveniles with mental and learning disorders or for those who required psychological or even psychotherapeutic support. Moreover, sick juveniles were stigmatized by prison doctors as malingerers and therefore often did not receive the necessary medical care. In this manner, prison doctors violated the medical ethical principle of beneficence.

## Data availability statement

The datasets presented in this article are not readily available because as the archival documents are the sources of this article, we are not authorized to provide copies to third parties. However, these documents can be accessed by researchers if they contact the archives directly. Following the rules of good scientific practice, we have indicated the signatures of the archival documents, ensuring the transparency of our research. Requests to access the datasets should be directed to OK, oxana.kosenko@uni-ulm.de.

## Author contributions

OK: Conceptualization, Data curation, Formal analysis, Investigation, Methodology, Resources, Validation, Visualization, Writing – original draft, Writing – review & editing. FS: Conceptualization, Formal analysis, Investigation, Methodology, Project administration, Resources, Supervision, Validation, Writing – review & editing.
